# Ultraprocessed food (UPF), health, and mechanistic uncertainty: What should we be advising the public to do about UPFs?

**DOI:** 10.1371/journal.pmed.1004439

**Published:** 2024-10-15

**Authors:** Eric Robinson, Alexandra M. Johnstone

**Affiliations:** 1 Department of Psychology, University of Liverpool, Liverpool, United Kingdom; 2 The Rowett Institute, University of Aberdeen, Aberdeen, United Kingdom

## Abstract

Eric Robinson and colleagues discuss what advice we can give in the face of the mechanistic uncertainty of ultra-processed foods.

## Teaser

In this perspective, we discuss why current mechanistic uncertainty on ultraprocessed foods (UPFs) and health acts as a major challenge to providing informed dietary guidelines and public advice on UPFs. Based on the balance of current evidence, we do not believe it is appropriate to be advising consumers to avoid all UPFs and we await further evidence to inform consumer guidance on the need to limit consumption of specifics foods based on their degree or type of processing.

There has been a recent surge in studies linking consumption of ultraprocessed foods (UPFs) to a broad range of negative health outcomes [1]. Most commonly defined and identified using the Nova classification system, UPFs make a considerable contribution to national diets, ranging from 15% to 58% as a proportion of daily energy across countries in a recent review [2].

This had led to calls for urgent public health action [3]. A small number of countries now include information explicitly about reducing intake of UPFs in their national dietary guidance [4]. Countries that do not currently include UPF in their national dietary guidance, such as the United Kingdom, are now reviewing evidence to determine the public health threat that UPFs pose [5].

Based on current evidence, we consider, what should we be advising the public to do about UPFs? In this perspective, we discuss why current mechanistic uncertainty on UPFs and health acts as a major challenge to providing informed dietary guidelines and public advice on UPFs.

The overwhelming majority of evidence linking UPFs to worse health is observational, where cause and effect are inferred. Observational studies are now numerous and appear to provide very consistent evidence that diets higher in UPFs are associated with a range of negative health outcomes (including obesity, noncommunicable diseases, and mortality), although strength of evidence varies by health outcome[1]. A widely accepted limitation of observational studies is that causal inference cannot be made, as unmeasured confounding has potential to explain associations observed. Recent work suggests that unmeasured confounding explaining UPF–weight gain associations in longitudinal cohort studies is at least plausible [6].

There is a paucity of direct causal evidence on UPFs and health or evidence of mechanism(s) from experimental studies [7], likely in part, because such studies can be time and cost intensive. There is some limited evidence from a small number of nonhuman and in vitro models that provide very initial backing for plausible theories on how some specific aspects of food processing could impact on biology in a way that would eventually be detrimental to health. Because of available evidence, it is therefore unclear what the mechanisms are that explain why diets higher in UPFs are associated with worse health in observational studies [7].

There is one widely cited randomised control trial on UPFs, energy intake, and weight gain [8]. A major limitation to the design of the study, as noted by the study authors, was that foods in the UPF condition (as opposed to the UPFs and beverages combined) served in the study had a different macronutrient profile to the non-UPFs and this resulted in a higher food energy density. Food energy density has a very strong effect on energy intake under laboratory conditions, and, therefore, one cannot disentangle with certainty the influence of macronutrient profile from level of processing in this study [9].

**Fig 1:**
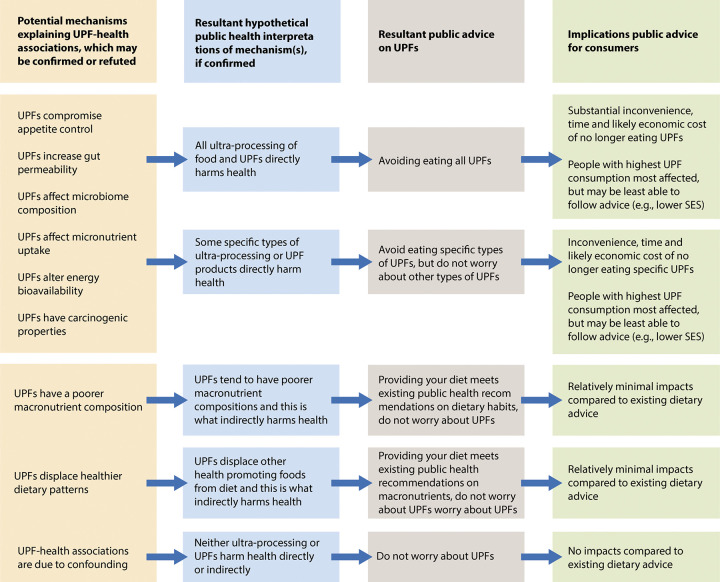
An illustration of how mechanistic uncertainty over UPF and health can have ramifications on the nature and implications of public dietary advice.

This study limitation highlights a broader issue—we do not know with any certainty ***why*** UPF consumption is associated with worse health in observational studies. A diet higher in UPFs may (or may not) be associated with worse health because it has a different macronutrient profile than a diet containing less UPFs. Other proposed potential mechanisms that could explain the association between UPFs and health are numerous (see Fig 1 for examples). A more extreme explanation of the association between UPFs and health outcomes in observational studies is that there is no actual “mechanism” at play, and, instead, the relationship is not causal. Conversely, some potential mechanisms would suggest that UPFs are directly problematic for health (e.g., carcinogens in some specific UPF ingredients or processing techniques), whereas others would suggest that UPFs may act indirectly on health (e.g., displacement of health promoting dietary patterns). To complicate matters, UPFs are a very wide and diverse collection of foods characterised by many different ingredients and processing techniques. Therefore, unless all types of UPF harm health, the specific mechanisms that explain UPF–health associations will dictate which specific UPF product types harm health (e.g., products with versus without specific types of emulsifiers).

This current level of uncertainty is a challenge because potential explanations for why UPF consumption is associated with worse health may have very different implications for public health and, therefore, what guidance and advice is given to the public. Implications could range from the public being told to avoid all UPFs, through to not worrying about UPFs at all, with other more nuanced and prescriptive messages between the two extremes (Fig 1).

Although there is substantial uncertainty at present, an argument based on the precautionary principle would be that immediate advice to the public should be given. However, there will be consequences of any public advice or national guidelines about UPFs. Because of the large role UPFs play in many people’s diets, avoiding all UPFs would cause substantial inconvenience, time, and/or economic cost. Those with the highest UPF consumption would be most affected, and many groups with higher UPF consumption (e.g., those in lower socioeconomic status (SES)) [10] may be least able to follow the advice because of social and economic circumstances. Different UPF dietary guidance could, therefore, have very different impacts on the public (Fig 1). At what point a precautionary principle is enacted and the public are provided with advice about UPFs also matters because science on UPFs and health is in its infancy and mechanistic uncertainty is high. Recently, a new wave of observational studies on UPFs have started to appear which suggest that some, but not all, types of UPFs in the diet may be associated with negative health outcomes [11]. These findings suggest that dietary advice to nondiscriminatively avoid all UPFs now commonly found in media coverage of the topic [12] is likely to be incorrect.

Getting dietary advice and policy on UPFs correct is, of course, important because of the burden of ill health that could be reduced, but also because providing premature and incorrect advice can cause unnecessary anxiety and mistrust of science. These considerations are particularly important in the context of UPFs, as for many people with more limited resources (e.g., less time, money, and/or a greater need for foods with longer shelf or use by dates), these food types invariably make life easier, and, therefore, their removal would have a “social cost.” Likewise, there may be unintended consequences of advice to avoid UPFs. Possible unintended consequences could include worsening of mental health among those who worry about their health or live with eating disorders, particularly if social circumstances make avoiding UPFs difficult. Alternatively, avoiding some types of UPFs could presumably in some contexts result in selection of non-UP alternatives that are higher in energy or macronutrients of concern. The potential health benefits of advising the public to avoid UPFs needs to be weighed carefully against mechanistic uncertainty and any likely negative social costs. Based on the balance of current evidence, we do not believe it is appropriate to be advising consumers to avoid all UPFs and we await further evidence to inform consumer guidance on the need to limit consumption of specifics foods based on their degree or type of processing.

We know with certainty that foods that are energy dense and/or high in saturated fat, salt, or sugar are detrimental to health, and we should continue to advise consumers to limit consumption of these foods. Mechanistic uncertainty over food processing and health should not prevent immediate and much needed public health policy to regulate the food industry in order to dramatically reduce the advertisement, availability, and dominance of foods high in energy and/or saturated fat, salt, or sugar on national diets. However, mechanistic uncertainty should determine how the public are communicated to and play a central role in determining public advice and emerging national dietary guidance on UPFs and food processing health risks.

## Disclaimer

The views expressed are those of the author(s) and not necessarily those of the NIHR or the Department of Health and Social Care.
